# Effect of an 8-Week Individualized Training Program on Blood Biomarkers, Adipokines and Endothelial Function in Obese Young Adolescents with and without Metabolic Syndrome

**DOI:** 10.3390/ijerph16050751

**Published:** 2019-03-01

**Authors:** Mohamed Sami Zguira, Maamer Slimani, Nicola Luigi Bragazzi, Meriem Khrouf, Faten Chaieb, Bernard Saïag, Zouhair Tabka

**Affiliations:** 1 Higher Institute of Sport and Physical Education of Gafsa, Gafsa 2100, Tunisia; sami-zguira@hotmail.fr; 2 Department of Physiology and Lung Function Testing, Faculty of Medicine Ibn-El-Jazzar, University of Sousse, Sousse 4000, Tunisia; fat.chaieb@gmail.com (F.C.); tabkazouhair@yahoo.fr (Z.T.); 3 Postgraduate School of Public Health, Department of Health Sciences (DISSAL), Genoa University, 16132 Genoa, Italy; maamer2011@hotmail.fr; 4 Department of Neuroscience, Rehabilitation, Ophthalmology, Genetics, Maternal and Child Health (DINOGMI), Section of Psychiatry, Genoa University, 16132 Genoa, Italy; 5 Hôpital Universitaire Fattouma Bourguiba, Cardiologie, Monastir 5000, Tunisia; mariemkhrouf@yahoo.fr; 6 Laboratory “Movement Sport and Health Sciences”, UFR APS University of Rennes 2, Avenue Charles Tillon, 35044 Rennes CEDEX, France; bernard.saiag3500@gmail.com

**Keywords:** individualized training program, diabetes and metabolic syndrome, obesity, blood biomarkers, adipokines, endothelial function

## Abstract

Obesity is a chronic condition whose incidence is growing due to lack of exercise and frequent nutrition disorders. Childhood obesity has reached epidemic proportions worldwide. One of the best treatment methods is physical training. However, conflicting results have been reported regarding its clinical effectiveness. These contrasting findings may be due to the type and intensity of the adopted physical training program. Therefore, the purpose of the current study was to investigate the effect of an 8-week individualized physical training program on endothelial function, blood biomarkers and adipokine levels in obese adolescents with and without metabolic syndrome (MS). One-hundred-and-twenty-two obese adolescents (71 obese without MS and 51 obese with MS) aged 14 ± 2 years were included in this study. The 8-week individualized training program decreased glucose, triglycerides, total cholesterol, low-density lipoprotein cholesterol and leptin in obese subjects with and without MS. However, adiponectin and endothelial-dependent vasodilatation increased in the follow-up study in both groups. Taken together, the findings suggest that individualized training program is an effective means for the treatment of obesity and MS in pediatric populations.

## 1. Introduction

Obesity is a chronic condition whose incidence is growing mostly due to lack of exercise and frequent nutrition disorders [1]. Childhood obesity has reached epidemic proportions worldwide. 

In Tunisia, the prevalence of obesity increased more than twofold during the last two decades to reach 22.7% in women, but only 6.4% in men [2]. In addition, cardiovascular risk is frequently associated with obesity in adults and children as well [3] through risk factors, such as increased blood glucose, fasting plasma triglycerides (TG), insulin levels, leptin concentration, low-density lipoprotein (LDL) cholesterol, and high-density lipoprotein (HDL) cholesterol [4,5]. Other risk factors, such as endothelial dysfunction and greater arterial stiffness, can also explain the development of cardiovascular risk [6,7]. 

In recent decades, new knowledge and new insights have been obtained on the mechanistic basis of endothelial homeostasis. Endothelial progenitor cells (EPCs) have been recognized as important factors of endothelial repair because they replace lost endothelial cells [5]. 

In this sense, Bruyndonckx et al. [8,9,10] reported an imbalance between endothelial damage and repair in obese adolescents. Second, brachial artery flow-mediated dilation (FMD) has been used extensively in clinical studies as a non-invasive assessment of endothelial function of large vessels, and it has been found to be particularly useful for pediatric cardiovascular risk evaluation since the last decades. Hence, acetylcholine (ACh) is the most common endothelial-dependent vasodilator used in this setting for performing endothelium-dependent and independent vasodilation. 

Metabolic syndrome (MS) has become one of the most important global public health problems, worldwide [11], together with diabetes and cardiovascular disease [12,13]. The prevalence of MS among obese children and adolescents is approximately 30% [14]. In addition, the risk for developing all of the features of MS during adult life is four times increased in children who were obese at seven years [15]. 

MS was first identified by Reaven as syndrome X and described as the coexistence of multiple metabolic derangements that included hyperinsulinemia, glucose intolerance, hypertension, decreased HDL cholesterol, and elevated TG. MS can be diagnosed when three or more of the following risk factors coexist: central obesity, elevated blood pressure, elevated fasting glucose, elevated TG and reduced HDL [16,17,18,19]. 

A better understanding of mechanisms underlying these health problems would facilitate the development and discovery of new methods/therapies for the proper treatment and management of obesity-associated MS. 

In this sense, one of the best treatment methods is physical training. However, conflicting results have been reported in the literature regarding its clinical effectiveness. Some studies showed a beneficial effect of physical training for the treatment of obesity and MS by decreasing glucose level, total cholesterol (TC) and LDL cholesterol [20,21,22,23]. In contrast, other studies found that physical training increased glucose level and LDL [24]. 

These contrasting findings may be due to the type and intensity of the adopted physical training program. Therefore, the purpose of the current study was to investigate the effect of an eight-week individualized physical training program on endothelial function, blood biomarkers and adipokine levels in obese adolescents with and without MS.

## 2. Materials and Methods 

### 2.1. Participants

One-hundred-and-twenty-two obese adolescents (71 obese without MS (Ob), and 51 obese with MS (ObWMS)) aged 14 ± 2 years were included in this study. All studied groups consisted of college and secondary school students. 

The study population was stratified according to the body mass index (BMI) as follows: obesity was defined in the case of a BMI ≥ 97th age- and gender-specific percentiles for adolescents younger than 16 years and BMI ≥ 35 for adolescents older than 16 years. 

History of familial or individual cardiovascular disease and treatments was collected for each participant. Subjects with a history of cardiovascular disease, hypercholesterolemia, liver disease, renal disease, or a current smoking habit were excluded. None of the subjects were using drugs or other therapy for obesity, and none had prior history of injury that would prevent daily exercise. Only adolescents motivated for the training were selected. 

Subjects were classified as having the metabolic syndrome if they met the following pediatric criteria: (i) serum triglycerides ≥110 mg/dL; (ii) fasting glucose concentration ≥110 mg/dL; (iii) waist circumference ≥90th percentile (sex- and age-specific) or ≥94 cm for males and ≥80 cm for females [25,26].

Participants, as well as their respective parents or legal guardians, were informed about the experimental procedures and their possible risks and benefits before the start of the study. Written, informed consent from legal representatives and/or participants was obtained before the beginning of the study. 

The study protocol was approved by the ethical committee of the Farhat Hached Hospital, Sousse, Tunisia (protocol number IRB00008931). This study was performed in accordance with the latest version of the Declaration of Helsinki.

### 2.2. Experimental Design

The testing period was divided into two visits: before and after training. The first visit served to complete a questionnaire (socio-demographic characteristics such as age, sex, diet, and physical activity). Anthropometric parameters and laboratory tests were also performed in fasting state. More specifically, the assessment of blood biomarkers, adipokines, and endothelial function were scheduled ≥72 h before each trial. 

Furthermore, participants also performed a six-minute walk test to assess their aerobic fitness. The same tests were performed ≥72 h after the trial.

### 2.3. Biochemical Assays

Blood samples were collected and drawn into ethylenediaminetetraacetic acid (EDTA)-containing tubes, chilled promptly in ice. Plasma and serum were separated immediately by centrifugation at 3100 rpm at 4 °C, and at 1000 rpm at room temperature for 10 min, respectively. Samples were stored at −80 °C until assay. 

At the time of analysis, routine chemical methods were used to determine serum concentrations of TC, HDL, TG, and glucose. The serum concentration of LDL was estimated using the Friedewald’s method [27].

### 2.4. Laser Doppler Flowmetry Combined with Iontophoresis of Ach and Temperature Elevation

Endothelium-dependent and independent vasodilation of the forearm skin microcirculation was quantitatively assessed by performing iontophoresis and skin heating combined with laser Doppler flowmetry (LDF) before and after a maximal test. More in detail, a laser beam penetrates the skin and a fraction of the light is backscattered by moving blood cells, undergoing a frequency shift according to the well-known Doppler principle. This results in the generation of a signal proportional to tissue perfusion.

Forearm skin blood perfusion was measured by means of a LDF apparatus (Periflux PF5001, Perimed, Stockholm, Sweden). The skin temperature was monitored throughout the trial and maintained constant at 32 °C, by the same LDF heating probe. The basal perfusion index was measured during the first five minutes at rest, without infusion of ACh and without heating the skin (skin temperature = 32 °C). Baseline skin blood perfusion was defined as the mean value recorded during 4 minutes. 

In order to investigate the endothelium-dependent vasodilation, iontophoresis of graduated doses of ACh was undertaken [28,29]. Iontophoresis is a noninvasive standard method of drug application that allows the local transfer of electrically charged substances across the skin by using a small electric current. The electrical potential difference actively causes ions in the solution to migrate according to their electrical charge. ACh (diluted at 2% solution) was used to fill the chamber of the electrode. 

In the present investigation, we used a delivery current of 10 mA and administered three successive doses of ACh for ten seconds with an interval of two minutes between each dose in order to achieve a plateau of the response following each delivery of ACh. Lastly, the laser probe was heated to 44 °C for five minutes, and we recorded the maximal response to local skin heating, i.e., the endothelium-independent maximal vasodilation. In order to eliminate baseline variability, the maximum skin perfusion value following iontophoresis was expressed as the maximum percent change from the baseline [28].

Perfusion index after the third dose of ACh iontophoresis was considered the maximal endothelial response (expressed in arbitrary units). Maximum perfusion index was measured after heat hyperhemia and without ACh infusion (skin temperature = 44 °C, arbitrary units) [28]. 

Results were computed and expressed as ACh-induced percentage changes in perfusion index versus basal values.

ACh chloride (Sigma-Aldrich, St Gallen, Switzerland) was obtained from a commercially available source, dissolved in deionized sterile water to 2% solution before the start of the experimental protocol and kept on ice at −4 °C.

### 2.5. Ventilatory Parameters and Lipid Oxidation Assay

Participants performed a five six-minute stage exercise at 20%, 30%, 40%, 50%, and 60% of their maximum theoretical workload (W_max_) [30]. 

We calculated the following theoretical values for each participant using the equations of Wasserman [31]: 

Maximum oxygen consumption (VO_2max_) = (52.8 × M) − 303.4 × W_max_= (VO_2max_ − 10(×M)) × (10.3) − 1 (M: body mass in kg). 

An electromagnetically-braked cycle ergometer (Ergoline, Bitz, Germany) was used for all tests. VO_2_ and VCO_2_ were measured breath-by-breath through a mask connected to O_2_ and CO_2_ analyzers (ZAN 600, Megeräte, Selb, Germany). 

Ventilatory parameters were averaged every 30 s during sub-maximal exercise testing. 

We calculated the maximum lipid-oxidation point (Lipoxmax), expressed in watts (W), which corresponds to the exercise intensity at which the highest rate of lipid oxidation is achieved (lipid oxidation at Lipoxmax, expressed in mg/min), according to the following equation [32,33]: 

Lipid oxidation (mg/min) = 1.6946 × VO_2_ − 1.7012 × VCO_2_, with VO_2_ and VCO_2_ expressed in mL/min.

### 2.6. Training Program

The training program was completed during the second trimester of the school year (from January to February 2012). The design of the individualized training intervention was based on our previous studies [32,33,34,35]. 

Briefly, the individualized training intervention was performed within the usual 90-min of supervised activity *per* day at a heart rate that corresponded to Lipoxmax, 3 days per week, during the 8-week intervention period. Exercise training intensity based on the participant’s heart rate corresponding to Lipoxmax was assessed at the first visit and was controlled by monitoring the heart rate with a sport-tester device (Vantage NV, Polar Electron, Kempele, Finland).

### 2.7. Statistical Analysis

Data were presented as mean values ± standard deviation (SD). Shapiro-Wilk’s test was used to determine the data’s normal distribution. This test was preferred to other tests for normality of data distribution given the small sample size of our population. 

Two-way repeated measures analysis of variance (ANOVA) (2 (group) × 2 (time)) was applied to test the main effects between pre- and post-test (time) and between the two groups (Ob versus ObWMS). The Bonferroni test was used as a post-hoc test correcting for multiple comparisons. Effect sizes (ES) were interpreted using the Hopkins’ rule of thumb: ES < 0.2 = trivial; ES in the range 0.2–0.6 = small; ES in the range 0.6–1.2 = moderate; ES in the range 1.2–2.0 = large; ES in the range 2.0–4.0 = very large; and ES > 4.0 = extremely large. Global ES (concerning the overall effect of the pre- and post-test control design) was also computed and interpreted accordingly.

All statistical analyses were carried out using the “Statistical Package for the Social Sciences” for Windows (SPSS Inc., Chicago, IL, USA, version 16.0).

All figures with *P*-values less than 0.05 were considered to be statistically significant, except where the Bonferroni test was applied, for ensuring protection against multiple testing.

## 3. Results

Before the training program, there were significant differences between Ob and ObWMS groups in terms of body mass, BMI and waist circumference (all, *P* < 0.001).

### 3.1. Effect of Training on Anthropometric Characteristics and Aerobic Capacity

Body mass (*P* = 0.02) and waist circumference (*P* < 0.0001) decreased in Ob group in the follow-up study. However, only waist circumference (*P* = 0.003) decreased in ObWMS group after training program (Table 1). 

**Figure 1:**
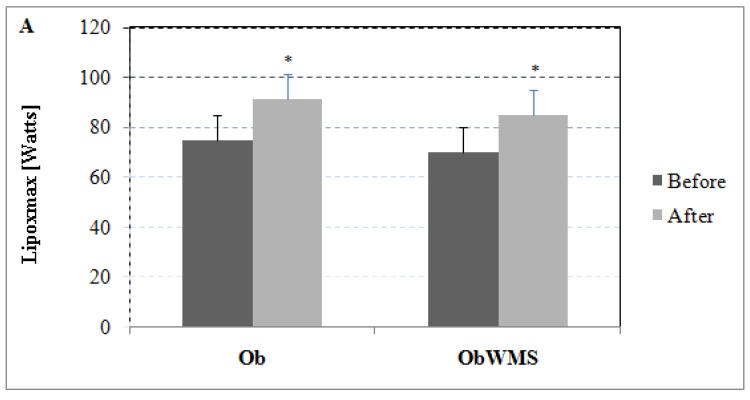

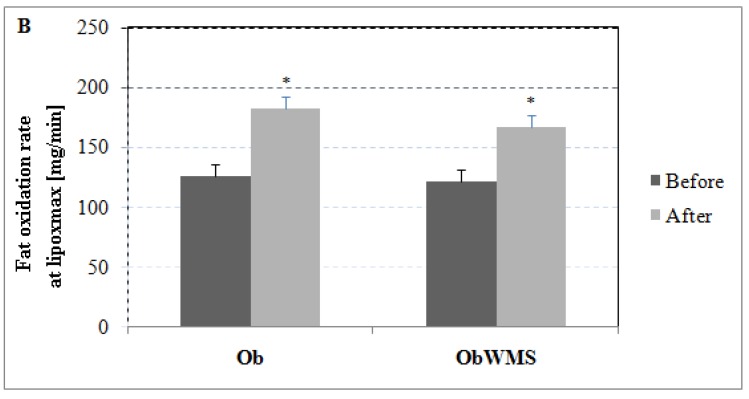
Effect of physical training on maximal lipid oxidation (Lipoxmax) during exercise: (**A**) Lipoxmax expressed in watts. (**B**) Rate of fat oxidation at Lipoxmax, expressed in mg/min. * pre vs. post training at *P* < 0.001, Ob: obese without metabolic syndrome, ObWMS: obese with metabolic syndrome.

Lipoxmax and rate of fat oxidation at Lipoxmax increased significantly in Ob (ES, 1.64, *P* < 0.0001; ES = 4.28, *P* < 0.0001, respectively) and ObWMS group (ES = 1.65, *P* < 0.0001; ES = 4.16, *P* < 0.0001, respectively), with greater improvements in Ob group (All, *P* < 0.001) (Figure 1).

### 3.2. Effect of Training on Blood Biomarkers and Adipokines

The 8-week individualized training program decreased blood glucose (7.4%, 4.4%), TG (11.8%, 9.7%), TC (15.1%, 9.7%), LDL (12.5%, 12.8%), and leptin levels (5.1%, 3.3%) in both Ob and ObWMS (all, *P* < 0.05), respectively. However, when considering the overall ES and taking into account the pre and post-test control-design, no significant differences between the Ob and ObWMS groups could be found for all measures (namely, glucose: ES = 0.35, *P* = 0.058, 95% CI −0.0027 to 0.1427; TG: ES = 0.17, *P* = 0.34, 95% CI −0.03310 to 0.09310; TC: ES = 0.08, *P* = 0.65, 95% CI −0.1694 to 0.2694; LDL: ES = 0.27, *P* = 0.14, 95% CI −0.0338 to 0.2338; and leptin: ES = −0.14, *P* = 0.44, 95% CI −2.3563 to 1.0363). 

Furthermore, the Ob group showed a significantly greater improvement in the adiponectin level (ES = 1.05, *P* < 0.0001, 95% CI 0.78 to 1.60) when compared with the ObWMS group (Table 2).

### 3.3. Effect of Training on Endothelial Function

**Figure 2:**
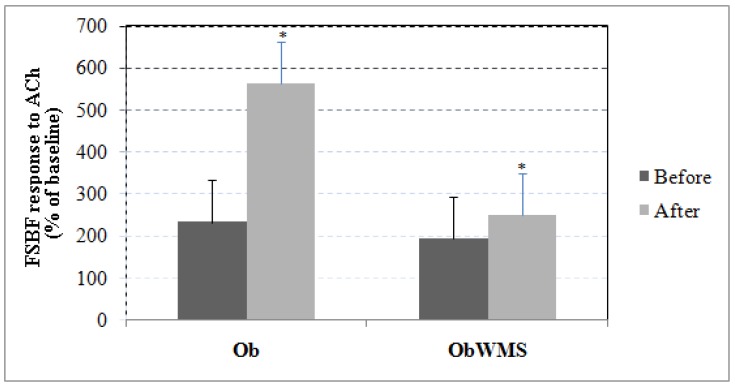
Effects of iontophoresis delivery of Ach on forearm skin blood flow (FSBF) before and after physical training in obese with and without metabolic syndrome. * pre vs. post training at *P* < 0.001, Ob: obese without metabolic syndrome, ObWMS: obese with metabolic syndrome.

Forearm skin blood flow (FSBF) response to Ach-induced endothelium-dependent relaxation increased significantly in the Ob and OBWMS groups (all, *P* < 0.001; ES = 4.49, ES = 0.92, respectively), with greater improvement in the Ob group when compared with the ObWMS group (ES = 4.05, *P* < 0.0001, 95% CI 249.412 to 298.588) in the follow-up study (Figure 2). 

**Figure 3:**
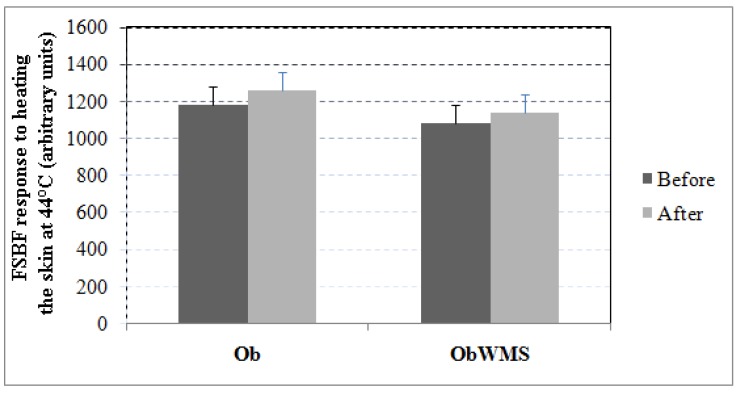
Forearm skin blood flow (FSBF) response to heating the skin at 44 °C (expressed as arbitrary units) observed before and after physical training in obese with and without metabolic syndrome. Elevation of the skin temperature at 44 °C, an endothelium-independent vasodilator, did not affect the forearm skin blood flow response of the groups significantly. Ob: obese without metabolic syndrome, ObWMS: obese with metabolic syndrome.

However, forearm skin blood flow response to heating the skin at 44 °C, an endothelium-independent vasodilator, remained unchanged in follow-up study in both Ob and ObWMS groups (All; *P* > 0.05) (Figure 3).

## 4. Discussion

This study examined the effect of an individualized training program on blood biomarkers, adipokines and endothelial function in obese adolescents with and without MS. The findings showed that an 8-week individualized training program was able to decrease blood biomarkers and increase adiponectin and endothelial function in both obese adolescents with and without metabolic syndrome. 

### 4.1. Effect of Training on Blood Biomarkers

The present findings showed that an eight-week individualized training program may induce small to moderate effect on blood biomarkers, in terms of glucose, TG, TC, and LDL in obese with and without metabolic syndrome. 

These findings are in line with the existing scholarly literature, namely with the results of Kim et al. [20], who reported that a six-week power training program decreased glucose level in obese Korean male adolescents. Meyer et al. [21] showed that six months of aerobic training reduced glucose, TC and LDL cholesterol in obese children. In contrast, other studies reported that glucose level and LDL increased [24] and remained unchanged [36,37,38,39,40] after physical training. 

As previously mentioned, this controversy between findings may be due to the mode, intensity, and volume of physical training involved. In this sense, a previous systematic review reported that physical training may induce beneficial effects on LDL cholesterol and TC, as well as with non-significant changes in glucose in adolescents who are overweight or obese [41]. 

In addition, a previous study showed that one-year aerobic plus resistance training was more effective in controlling metabolic syndrome, by improving TC, waist circumference, glucose, and adiponectin than aerobic training alone in obese adolescents [42]. However, regarding the difference between obese with and without MS groups follow-up study, similar results were reported. This suggests that an individualized training program is effective to the control and treatment of obesity and metabolic syndrome in pediatric populations. 

### 4.2. Effect of Training on Adipokines

Individualized training program increased adiponectin level in both obese with and without metabolic syndrome, with greater improvement in the obese group. However, conflicting results have been reported in the literature. Some studies showed a significant increase of plasma adiponectin after exercise training. Balagopal et al. [43] investigated the effect of three months of aerobic exercise on adiponectin levels on obese adolescents. The authors reported a positive effect of aerobic exercise on plasma adiponectin, by showing its increment from 4.44 ± 0.47 to 5.95 ± 0.49 µg/mL. 

Another study reported that 6 weeks of jump rope exercise training, five days per week, increased adiponectin levels in obese Korean male adolescents [20]. In this sense, Tjønna et al. [44] showed that three months of twice weekly high-intensity exercise sessions increased adiponectin level in overweight adolescents. On the other hand, Nassis et al. [36] reported no significant change in adiponectin level following 12 weeks of aerobic training in obese girls. 

Regarding the leptin level after physical training, some studies showed that long-term exercise training (i.e., 12 weeks, 7 months) decreased blood levels of leptin in obese individuals [45,46,47]. Accordingly, Gutin et al. [48] studied the effect of four months of aerobic training on leptin level in obese children. They showed the reduction of leptin concentrations, which subsequently increased following four months of training and detraining, respectively. This reduction may be due to the decrement of body fat and negative energy balance resulting from exercise training. In contrast, other studies reported no significant changes in leptin concentration after nine weeks of aerobic training in obese women [49], which is in line with the present findings. 

### 4.3. Effect of Training on Endothelial Function

Concerning the endothelial function, FSBF response to ACh-induced endothelium-dependent relaxation increased significantly following eight weeks of the individualized training program in both obese with and without metabolic syndrome groups with greater improvement in the obese group. Accordingly, Cohen et al. [50] reported that 14 months of a resistance training program improves endothelial dependent vasodilation in adults with type 2 diabetes. However, several mechanisms may explain the improvement of endothelial function or markers of endothelial function. First, vascular improvement was associated with improvement in blood glucose control. Second, the repetitive exposure to exercise may lead to provoke blood flow through the vessels supplying oxygen to the working muscles, which, in turn, increases shear stress [51]. In this sense, increased shear stress results in up-regulation of the nitric oxide (NO) and bioavailability [52] that may improve vasomotor function as well as inhibit atherogenesis [53,54]. In addition, exercise training improved endothelial function with an increase of NO biodisponibility and/or release, an increased functional capacity of circulating angiogenic cells and a decreased oxidant stress [40]. In contrast, Middlebrooke et al. [55] reported that six months of aerobic training have no effect on endothelial function in individuals with type 2 diabetes. Hence, a number of variables, such as training duration, intensity, and frequency, may account for the beneficial effect of physical training on endothelial function in individuals who are obese.

## 5. Conclusions

The present study showed that an eight-week individualized training program decreased glucose, TG, TC, LDL and leptin in obese with and without MS. However, adiponectin and endothelial dependent vasodilator increased in the follow-up study in both groups. This suggests that individualized training program is an effective means for the treatment of obesity and MS in pediatric populations. On the other hand, BMI did not decrease significantly in the study in one of the groups. Given this limitation, further studies, using larger sample sizes, are warranted.

## Figures and Tables

**Table 1:** Participants’ characteristics.

Parameter	Ob		ObWMS	
	Before	After	Before	After
Age (years)	13.3 ± 1.5		13.6 ± 1.2	
Body mass (kg)	71.1 ± 7.6	68.2 ± 7.7 *	90.1 ± 16.1 #	86.7 ± 16.5
Height (m)	1.63 ± 0.06		1.64 ± 0.05	
BMI (kg·m^−2^)	27.8 ± 2.1	27.2 ± 2.2	32.8 ± 5 #	31 ± 5.1
WC (cm)	93.7 ± 5.6	89.7 ± 5.8 **	106.5 ± 5.2 #	103.4 ± 5.3 *

Abbreviations: BMI: body mass index, Ob: obese without metabolic syndrome, ObWMS: obese with metabolic syndrome, WC: waist circumference, * pre vs. post training at *P* < 0.05, ** pre vs. post training at *P* < 0.0001, # Ob vs. ObWMS at *P* < 0.0001.

**Table 2:** Mean values and standard deviations (SD) of blood biomarkers and adipokines before and after the training program.

Parameter	Ob (n = 71)				ObWMS (n = 51)				Overall *P*
	Before	After	ES	*P*	Before	After	ES	*P*	
Glucose (mmol/L)	4.74 ± 0.22	4.50 ± 0.18	−1.20	<0.0001	5.11 ± 0.15	4.94 ± 0.26	−0.80	0.0001	0.058
TG (mmol/L)	1.41 ± 0.16	1.30 ± 0.17	−0.67	0.0001	1.79 ± 0.25	1.71 ± 0.12	−0.42	0.040	0.34
TC (mmol/L)	4.09 ± 0.52	3.61 ± 0.61	−0.85	<0.0001	4.42 ± 0.55	3.99 ± 0.78	−0.64	0.001	0.65
LDL (mmol/L)	2.35 ± 0.5	1.99 ± 0.32	−0.86	<0.0001	2.67 ± 0.4	2.41 ± 0.2	−0.83	<0.0001	0.14
Adiponectin (µg/mL)	2.25 ± 0.95	4.43 ± 1.48	1.76	<0.0001	2.56 ± 0.9	3.55 ± 1.14	0.97	<0.0001	<0.0001
Leptin (ng/mL)	21.99 ± 5.15	19.23 ± 3.3	−0.64	0.0002	26.51 ± 4.92	23.09 ± 5.54	−0.65	0.001	0.44

ES: effect size, Ob: obese without metabolic syndrome, ObWMS: obese with metabolic syndrome.
